# Green synthesis and characterization of copper nanoparticles for investigating their effect on germination and growth of wheat

**DOI:** 10.1371/journal.pone.0269987

**Published:** 2022-06-21

**Authors:** Humaira Kausar, Ansar Mehmood, Rizwan Taj Khan, Khawaja Shafique Ahmad, Sajjad Hussain, Fahim Nawaz, Muhammad Sajjad Iqbal, Muhammad Nasir, Tariq Saif Ullah

**Affiliations:** 1 Department of Botany, University of Poonch Rawalakot, Azad Kashmir, Pakistan; 2 Department of Botany, the University of Azad Jammu and Kashmir (UAJK), Muzaffarabad, Pakistan; 3 Department of Agronomy, MNS University of Agriculture Multan, Punjab, Pakistan; 4 Department of Botany, University of Gujrat, Punjab, Pakistan; 5 Department of Botany, University of Kotli, Azad Jammu and Kashmir, Pakistan; Aligarh Muslim University, INDIA

## Abstract

Today, different types of nanoparticles (NPs) are being synthesized and used for medical and agricultural applications. In this study, copper nanoparticles (CuNPs) were synthesized using the aqueous extract of mint (*Mentha longifolia* L.). For the characterization of CuNPs, UV-visible spectroscopy, scanning electron microscopy, X-ray diffraction, and Fourier transform infrared spectrometry were used. The UV-Visible absorption peak at 558 nm confirmed the formation of CuNPs. The XRD pattern confirmed the phase-centered crystalline nature of CuNPs. FTIR analysis showed the O-H, Cu-H and C-C bonds, indicating the active role of these functional groups as reducing agents of Cu ions to CuNPS. The synthesized NPs were found to have an almost spherical shape with an average size of 23 nm. When applied to wheat, a condition dependent effect of CuNPs was found. Variety 18-Elite Line 1, Elite Line 3, and 18-Elite Line 6 showed maximum germination and growth rate at 50 mg CuNPs/L, while variety 18-Elite Line 5 showed that increase at 25 mg CuNPs/L. Beyond these concentrations, the seed germination and growth of wheat declined. In conclusion, the application of CuNPs showed a beneficial effect in improving the growth of wheat at a certain concentration.

## Introduction

Research in nanotechnology has evolved rapidly over the past few decades. Nanotechnology is a branch of science that deals with the atomic and molecular analysis of material objects [1]. Nanoparticles (NPs) are classified as particles up to 100 nm in size and consisting of chemical and physical processes, including unique properties [2]. They have a very significant role in various applications because of their unique properties such as optical, electrical, catalytic, electromagnetic, and mechanical, which enable them to be used in various sectors such as medical diagnostics, therapy, electronics, clothing, and agriculture [3]. Nano research is having an impact on a variety of fields, including the environment and catalysts [4–7]. Metal NPs production has been given great attention in the last few years, particularly to control and discover their possible applications and specific properties. The increased production of metal NPs has also increased their discharge into our environment, and the effect of their particular physical and chemical properties on the ecosystem is becoming a key concern [8]. NPs can have both negative and positive effects on the growth and development of a plant, and their influence on plants depends on the size, shape, and properties of both plant species and NPs [9].

Copper (Cu), an element in block D of the periodic table, is a microelement essential for plant improvement and growth. It is a cofactor of superoxide phenol oxidases, ascorbate oxidase, a part of regulatory proteins, and is involved in the electron transport chain during photosynthesis and respiration [10, 11]. Cu in the form of nanomaterial gains exceptional properties like its small size and high surface area, which give it chemical reactivity, physical resistance, magnetism, and optical effects [12]. Due to these unique properties, copper nanoparticles (CuNPs) are used for a wide variety of applications, including bioactive coatings, air and liquid filtration, sensors, ceramics, films, skin products, lubricant oils, inks, wood protection, and textiles. Recent estimates suggest that the global production of CuNPs, or Cu-based NPs, has come to 200 tons/year [13]. Their extensive engineering and use have led them to enter the environment and interact with agriculture. They can cause either positive or negative effects following contact with plants, depending upon the concentration of the NPs and the type of plant. It is therefore the need of the hour to investigate the effect of CuNPs on crops, as there are only a few studies available that show the influence of CuNPs on crop plants.

CuNPs can be engineered by way of specific routes like physical, chemical, and biological techniques [14–16]. The main problems with the physical and chemical methods are the time-consuming nature of them and their usage of exclusive and toxic chemicals [17]. The chemical methods are eco-incompatible, costly, and also have a low yield. In contrast to physical and chemical methods, biological methods (green synthesis) are more advantageous in the sense of being eco-friendly, cost-effective, and high yielding [18]. Green synthesis is largely motivated by environmental concerns, with the goal of developing a green pathway for NPs synthesis that is also contamination-free [19].

Green synthesis involves the use of algae [20], sea cucumbers [6], marine animals [21], plants [16, 22, 23], and microorganisms among many others [24, 25]. Biological techniques make it easier to reduce dissolved metal ions to a zero-valence state and produce the corresponding nanoparticles because of their inherent capabilities. NPs synthesis with the aid of using plants is beneficial over the use of microorganisms because it gets rid of the complicated procedure of keeping cellular cultures and can also be well scaled up [26]. Keeping in mind the importance and benefits of green synthesis, CuNPs were synthesized in this study by using a plant extract and their effect was evaluated on the growth and development of wheat (*T*. *aestivum*), an essential and widely consumed staple food. In Pakistan, wheat crops provide a living for 80 percent of farmers. It is also a very significant single commodity in Pakistan’s rural areas as a source of earnings [27]. Although some studies have reported the effect of CuNPs on wheat, they have used either very small concentrations (0.2, 0.4, 0.6, 0.8, and 1.0 ppm) of CuNPs [28] or chemically engineered CuNPs (50 nm) of large size [29]. In contrast to these, we used biologically synthesized CuNPs of 23 nm in size at a concentration of 0, 25, 50, and 100 mg/L.

Therefore, the first aim of this study is the green synthesis and characterization of CuNPs. In this study, an aqueous extract of *Mentha longifolia* will be used for the reduction of Cu ions to CuNPs at room temperature. The CuNPs will be characterized by UV-visible spectroscopy, scanning electron microscopy, X-ray diffraction, and Fourier transform infrared spectrometry. The second aim is the investigation of the effect of biosynthesized CuNPs on germination, growth, and biochemical attributes of the widely consumed staple food wheat.

## Material and methods

### Synthesis and characterization of CuNPs

A biological method, i.e., the use of an aqueous extract of *M*. *longifolia* was adopted in this study for the synthesis of CuNPs. The plant material was collected from the Rawalakot region of Azad Jammu and Kashmir, Pakistan. The plant was identified with the help of the flora of Pakistan and other available literature, and a voucher specimen was submitted to the herbarium, Department of Botany, University of Poonch Rawalakot. To prepare the aqueous extract, the whole plant was dried in the shade, ground to powder, and 25 g of powder was dissolved in 400 mL of distilled water, then kept at room temperature for 24 hours. After that, it was filtered through Whatman filter paper no. 42 and the filtrate was used for the synthesis of CuNPs. The CuNPs synthesis was attained by treating 80 mL of 1 mM CuSO_4_ at room temperature with 20 mL of plant extract, held in the dark, and color changes were observed. When the brown color developed (after 24 hrs), the reacting mixture was analyzed for UV-vis spectroscopy in the range of wavelengths between 300 to 800 nm using Perkin-Elmer lambda 750 spectrophotometers, indicating the formation of CuNPs. After initial indication, the reacting solution was centrifuged for 15 min at 10,000 rpm, the supernatant was discarded, the pellet was re-dispersed in distilled water and the centrifugation cycle was repeated at the same pace and duration. The final round of re-dispersion and centrifugation was accomplished with acetone to obtain cleaned and purified CuNPs. The morphology, phase, and capped functional groups of CuNPs were determined through scanning electron microscopy (MIRA 3 Tescan), x-ray diffraction analysis, and Fourier transform infrared spectrometry (Perkin Elmer Spectrum 100 FTIR).

### Application of CuNPs on wheat using Petri plate assay

#### Seed source, treatments, and germination

The seeds of four wheat varieties like 18-Elite Line 1, 18-Elite Line 3, 18-Elite Line 5, and 18-Elite Line 6 have been obtained from the Department of Plant Breeding Genetics, University of Poonch Rawalakot. The seeds were sterilized for 1 minute with 75 percent ethanol and 15 minutes with 2.5 percent calcium hypochlorite after being soaked in 0.6 percent HNO_3_ for 10 minutes to end seed dormancy for successful germination [30]. The experiment was conducted in a completely randomized design (CRD) with 5 replicates. The four concentrations of CuNPs (0, 25, 50, and 100 mg/L) were applied in two ways, i.e., seed treatment and foliar spray. For seed treatment, the seeds were soaked in an aqueous solution of CuNPs for 3 h, and then 10 seeds of each variety were evenly placed in each Petri plate (labeled) already containing wet blotting paper. All the Petri plates were placed at room temperature and allowed to germinate with a daily supply of 5 mL of each concentration as a foliar spray. Samples were collected after 10 days of treatment and examined the effects of CuNPs on germination, growth, and biochemical attributes.

#### Germination indices measurements

The germinated seeds were counted in each treatment for the measurement of germination percentage (GP). The GP was determined as GP = GN/SN × 100, where GN and SN are the total germinated seeds and tested seeds, respectively. The germination index (GI) was measured as GI = number of seeds germinated seeds/days of the first count + number of germinated seeds/days of the final count.

#### Growth parameters measurements

To estimate growth, the primary root and shoot length of 10 days of seedlings were measured. For this, 5 plants were selected randomly from each treatment, the length was measured in cm, and the average was calculated. Similarly, the fresh weight of seedlings was measured in grams with the help of an analytical balance.

#### Biochemical contents measurements

For the extraction of chlorophyll and carotenoid pigments, fresh leaves weighing 0.1 g were homogenized in 10 mL of 80% acetone. The homogenate was then centrifuged at 15000 rpm for 10 min, the pellet was discarded, and the absorbance of the supernatant was recorded at 666, 653, and 470 nm on the Shimadzu UV-1601 spectrophotometer. The formulae developed by [31] were used for the calculation of chlorophyll and carotenoid: Chla (mg/g) = [(12.25 x A663.2)—(2.79 x A646.8)] × ml acetone / mg leaf tissue, Chlb (mg/g) = [(21.50 x A646.8)—(5.10 x A663.2)] × ml acetone / mg leaf tissue. Total Chl = Chla + Chlb and Carotenoids (mg/g) = (1000 A470–1.8Chla– 85. 02 Chlb) /198. Total phenolic content was measured by Folin–Ciocalteu’s phenol reagent assay [32]. A 0.2 mL of plant extract mixed with 5 mL of 10-fold diluted Folin–Ciocalteu’s phenol reagent was kept for 4 minutes and then it was aided with 4 mL of sodium carbonate (7.5 percent w/v). The whole mixture was diluted up to a volume of 15 mL with distilled water and mixed well. The reaction was permitted to stand for 90 min, and the absorption of each sample was reported at 760 nm using a spectrophotometer Shimadzu UV-1601. The total phenols were measured using the calibration curve for gallic acid (GA) and presented as the equivalent of mg GA / g FW. To extract total soluble sugars, 50 mg of fresh leaves were crushed thoroughly in 5 mL of 80% ethanol and centrifuged for 10 minutes at 3000 rpm. From the supernatant, 0.1 mL was taken in a test tube and kept in a water bath for the evaporation of ethanol. After the ethanol had completely evaporated, 4 mL of 0.2 percent enthrone reagent was added to the test tube, heated in boiling water for 10 minutes, and then allowed to cool at room temperature. The absorption of each sample was reported at 760 nm using a spectrophotometer Shimadzu UV-1601. Total soluble sugars were measured using the calibration curve for glucose and presented as the equivalent of mg Gu/g FW.

### Statistical analysis

The experiment was conducted in CRD using three replicates and analysis of variance (ANOVA) was performed in statistix 8.1. The statistical difference between means was evaluated, considering p < 0.05 as a significance level.

## Results and discussion

### Synthesis, morphology, size, and structure of CuNPs

**Fig 1:**
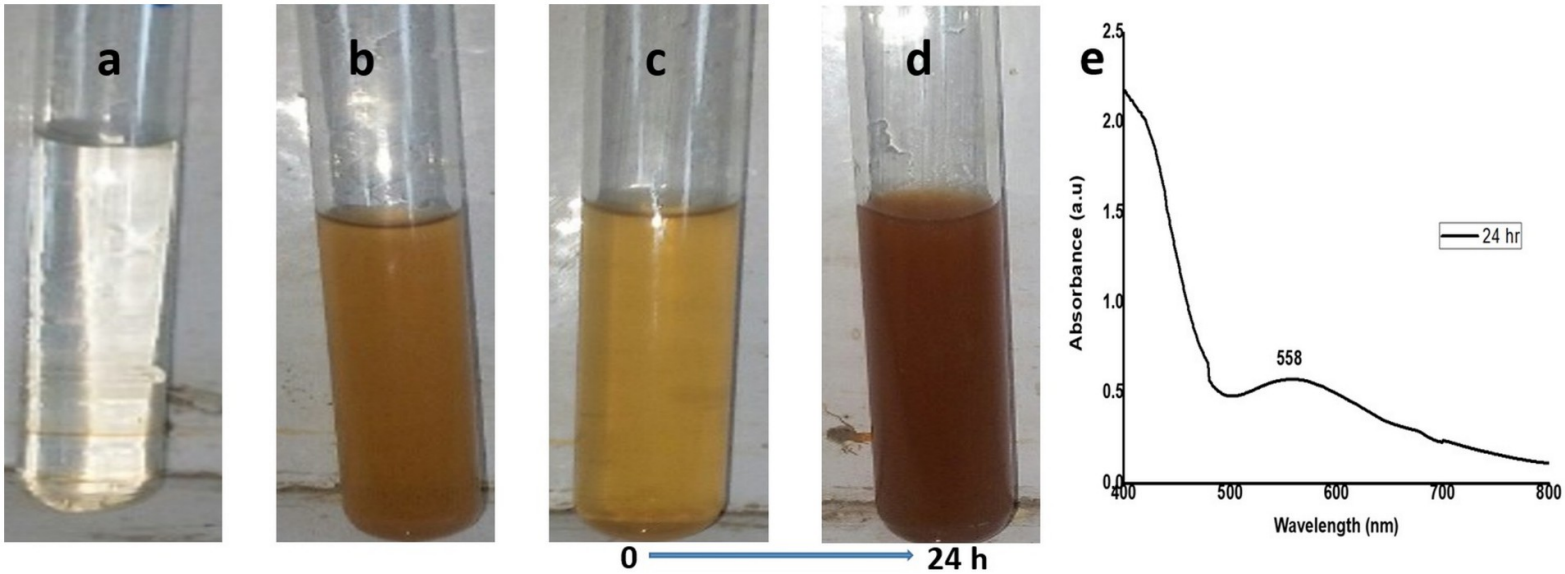
Synthesis of CuNPs. a copper sulfate solution. b plant extract. c mixture of copper sulfate solution and plant extract at zero-time. d change in color of solution after 24 h of time lap. e UV-visible spectrograph of CuNPs.

**Fig 2:**
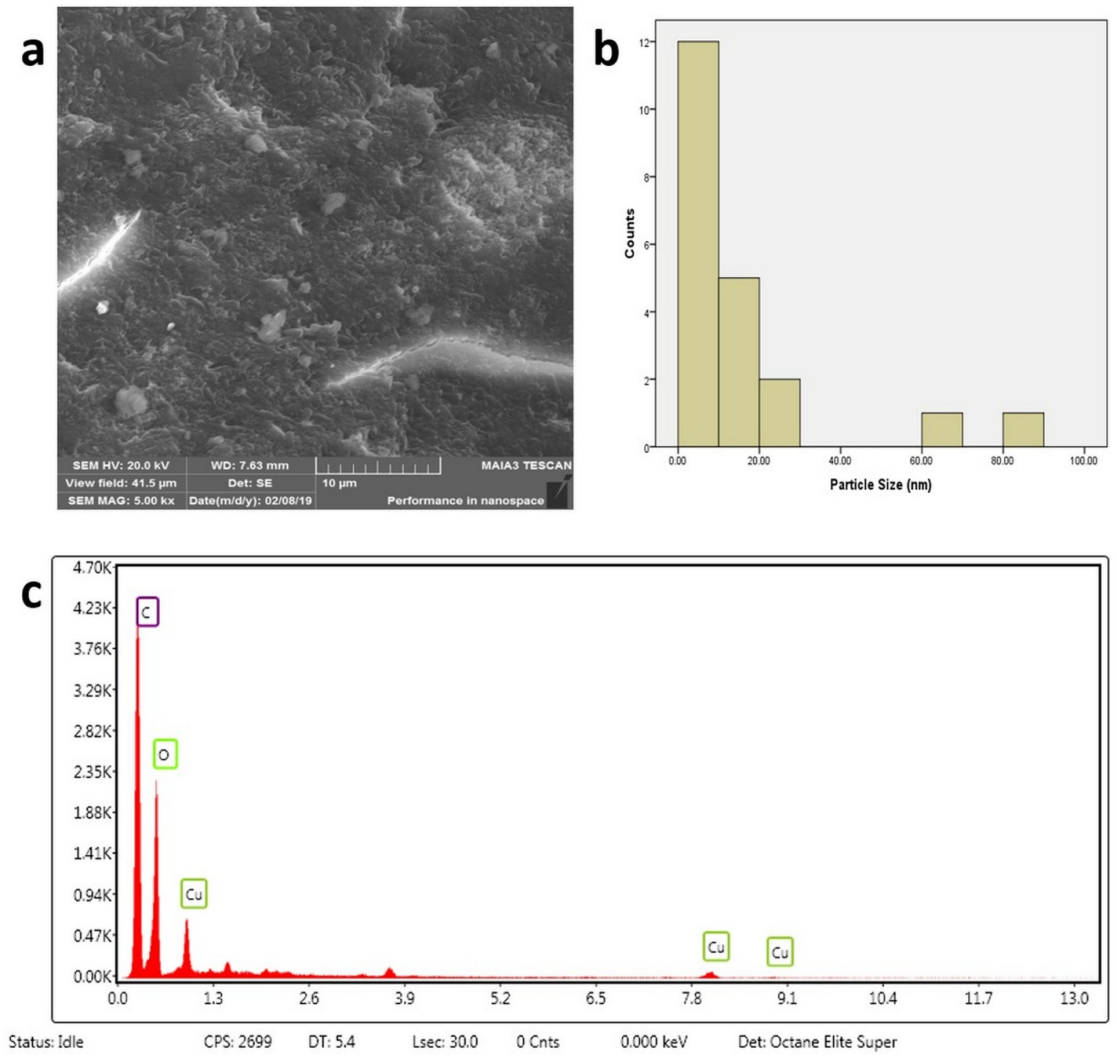
Characterization of CuNPs. (a) FESEM micrograph of CuNPs, showing spherical shape of CuNPs; (b) Size distribution histogram of CuNPs, showing average size of 23 nm. c EDX spectrum of CuNPs.

**Fig 3:**
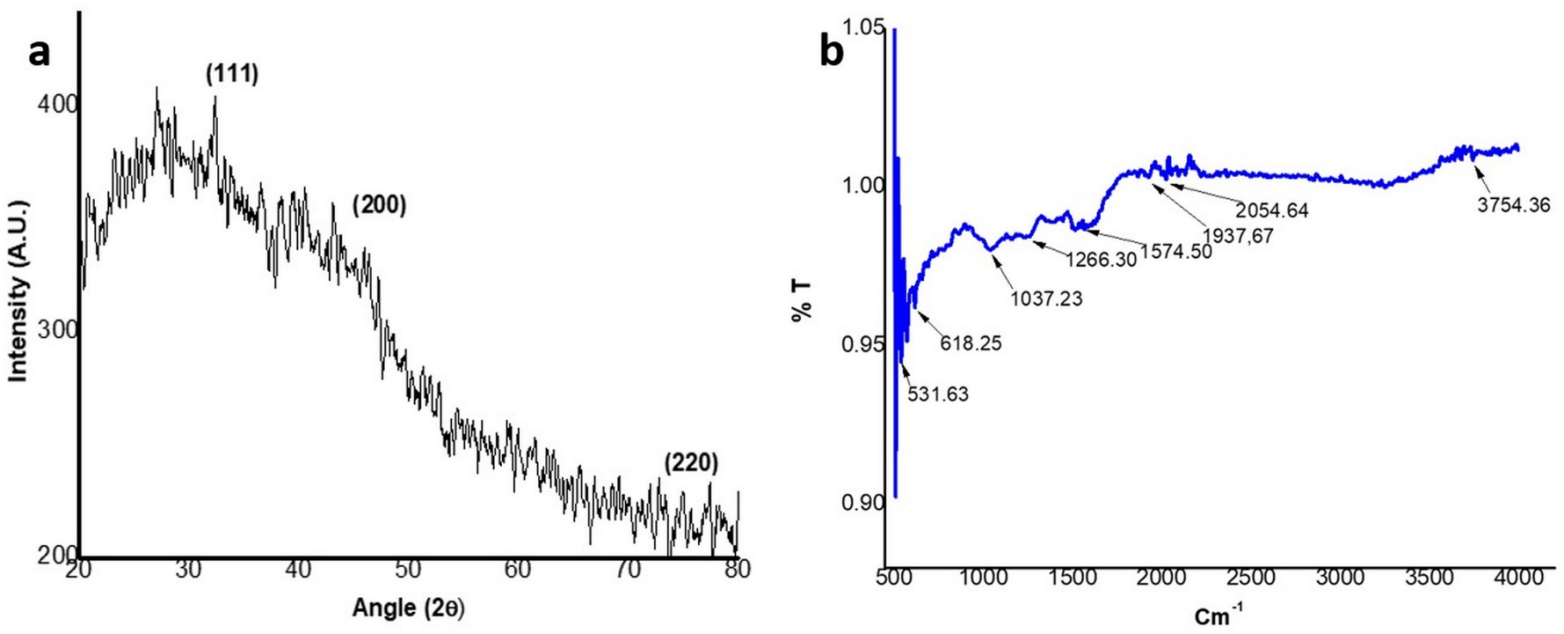
Characterization of CuNPs. (a) XRD pattern of CuNPs, predicting crystalline nature of CuNPs; (b) FTIR spectrum of CuNPs, confirming functional groups from plant extract capping the CuNPs.

The CuNPs were synthesized from the aqueous extract of *M*. *longifolia*. The addition of aqueous extract to the aqueous solution of CuSO_4_ resulted in the development of a color change from pale yellow to dark brown over the course of 24 h, the first indication of the formation of CuNPs in the solution (Fig 1). This color change is developed due to the excitation of surface plasmon resonance (SPR) [33]. Previous research has also suggested that brown color can be established in the reaction mixture of plant extract and CuSO_4_ [34]. This color change was further accomplished by taking a UV-vis spectrum of a brown-colored solution in the range of 400 to 800 nm (Fig 1). The spectrophotometric study revealed an absorption band at 558 nm, a result of interaction between the free electrons presents on the surface of metal NPs and light of specific wavelengths. This absorption band indicates the formation of CuNPs in the colloidal solution because it is a known fact that CuNPs show characteristic absorption peaks in the range of 200–800 nm [35]. Moreover, it has been analyzed that a single SPR band represents round-shaped nanoparticles, whereas two or more SPR bands correspond to the anisotropy of nanoparticles [36]. In our study, CuNPs in the solution showed a single SPR band, which reveals the round shape of CuNPs. The morphology and size of the prepared CuNPs are shown in the images of SEM (Fig 2a) and their mean size distribution histogram (Fig 2b). The CuNPs exhibited a spherical shape, were distributed irregularly, and had an average size of 23 nm. Fig 2c represents the energy dispersive x-ray (EDX) of the powder and shows the presence of Cu in the material. The peaks belonging to elemental Cu, C, and O were clearly detected, and there were no extra peaks, demonstrating the purity of the synthesized NPs and correlating with the XRD analysis. It’s also possible that the existence of C and O is due to bioactive molecules that have been capped. Furthermore, only a few copper atoms on the surface of the NPs are likely to have been oxidized, yielding modest quantities of CuO and Cu_2_O. The XRD pattern of CuNPs is shown in Fig 3, confirms the face-centered crystalline nature, as all the peaks match Cu with FCC symmetry and are consistent with JCPDS No: 04–0836, which reveals the crystalline nature of CuNPs [37]. With the help of FTIR (Fig 3), the functional groups of all the possible biocompounds involved in the reduction and capping of CuNPs are identified. The FTIR spectrum showed peaks at 3754.36, 2054.64, 1937.67, 1587.34, 1266.30, and 1043.06 cm^-1^. The peak at 3754.36 cm^-1^ corresponds to O-H stretching frequency of hydroxyl groups of polyphenols. The peaks at 2054.64 and 1937.67`cm^-1^ correspond to Cu-H (metal-hydrogen) bonds. The peaks at 1587.34, 1266.30, and 1043.06 cm^-1^ could be due to C-C stretching vibrations of an aromatic compound, C-O stretching of carboxylic acid, and ester bonds of phenolic compounds. These results suggest an interaction between the functional groups present in plant extract and Cu ions that results in bio-reduction, the formation, and stabilization of CuNPs [4, 6, 38]. CuNPs are thought to be formed from copper salts using plant extracts in three steps: production of copper ions, reduction of the ions, and lastly, oxidation of the reduced ions [16, 20].

### Effect of CuNPs on seed germination of wheat

**Fig 4:**
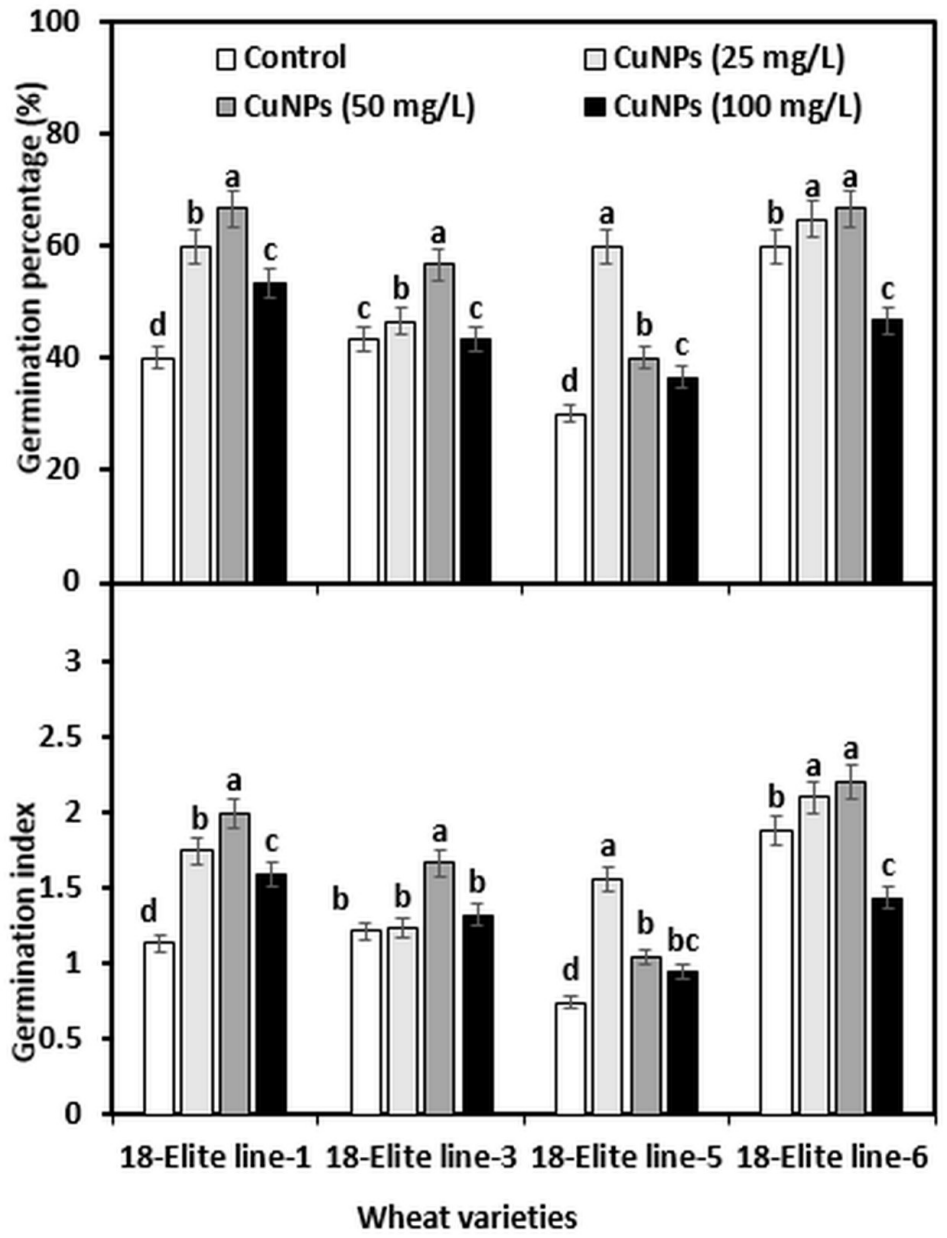
Seed germination percentage and germination index of wheat varieties affected by different concentration of CuNPs. Different letters in the same sub-group of columns indicate significant difference at p < 0.05 level.

Seed germination is the beginning of a physiological process of a plant that needs water imbibition and is directly linked to the yield of the plant. In this study, seed germination and germination index of the tested wheat varieties exhibited an almost similar trend, except for the variety 18-Elite line-5. Fig 4 shows the GP and GI of wheat varieties after the 10^th^ day of treatment with different concentrations of an aqueous solution of CuNPs. When exposed to 50 mg CuNPs/L, GP and GI increased considerably in varieties 18-Elite line-1, 18-Elite line-3, and 18-Elite line-6, while the variety 18-Elite line-5 showed increased GP and GI under 25 mg CuNPs/L. After 50 mg CuNPs/L exposure, the GP increased to approximately 67, 31, 33, and 11 percent in 18-Elite line-1, 18-Elite line-3, 18-Elite line-5, and 18-Elite line-6, respectively, of the GP observed in control. However, in 18-Elite line-5, a higher increase was found at 25 mg CuNPs/L (100%) as compared to control. A similar trend was found for GI, it increased to approximately 76, 37, 41, and 17 percent in 18-Elite line-1, 18-Elite line-3, 18-Elite line-5, and 18-Elite line-6, respectively, of the GI, recorded in control after the application of 50 mg CuNPs/L. More than double the increase in GI was apparent in 18-Elite line-5 after treatment with 25 mg CuNPs/L. When varieties were exposed to 100 mg CuNPs/L, no statistical difference was found in GP compared to control, except in variety 18-Elite line-6, which showed a reduced GP and GI as compared to control. Similar results were reported by [39], where lettuce seeds showed better germination when treated with CuNPs. It is found that NPs tend to come into contact with seed coats, penetrate the seeds, and improve seed germination and seedling growth of the plants [40]. They found that CuNPs penetrated the cell wall and formed new pores, so water absorption was improved, and as a result, favorable seed germination was started. Another study demonstrated that high seed germination rates might be due to the photo-generation of active oxygen like hydroxide anions and superoxide, which causes re-activation of aged seeds. In the nanocomposite, activated carbon provides moisture and a large surface area for seed germination [41].

### Effect of CuNPs on seedling growth of wheat

**Fig 5:**
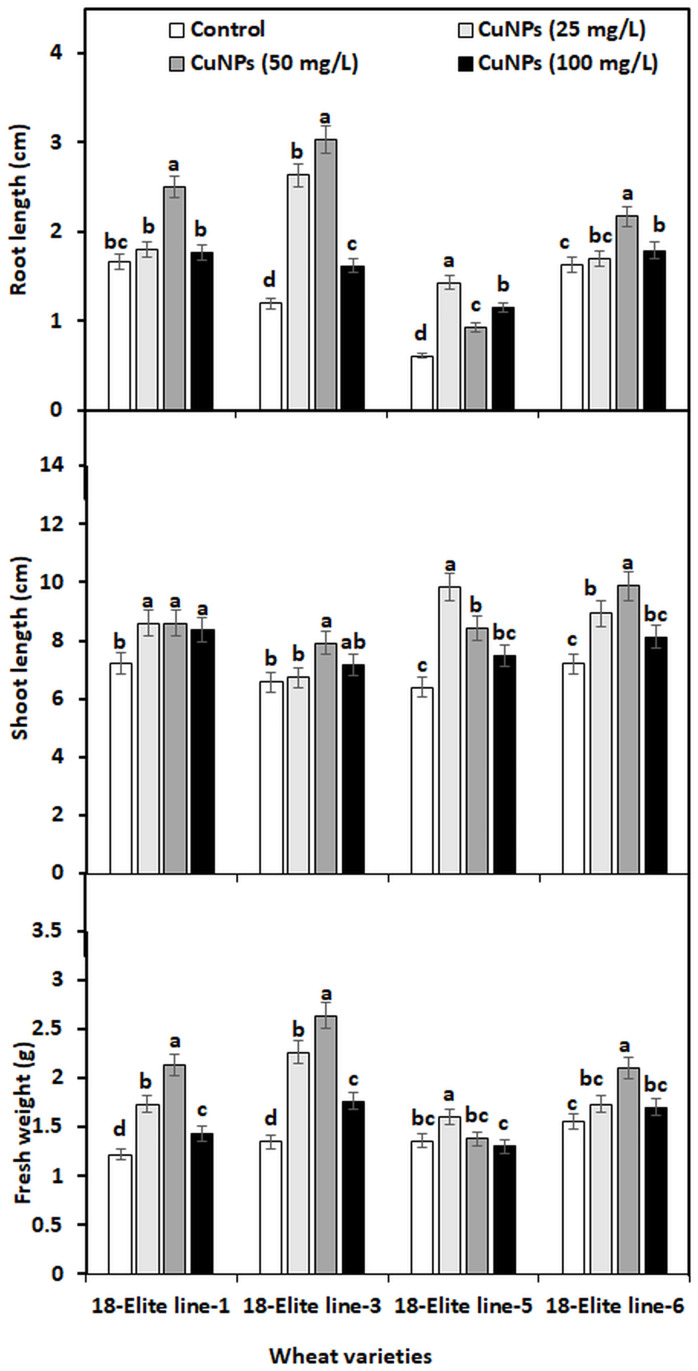
Root length, shoot length, and fresh weight of 10 days wheat seedlings affected by different concentration of CuNPs. Different letters in the same sub-group of columns indicate significant difference at p < 0.05 level.

RL, SL, and FW were measured for all the tested varieties after 10 days of seedling (Fig 5) to investigate the effect of CuNPs on seedling growth of wheat. All the concentrations of CuNPs posed a positive effect on the RL of all wheat varieties, but the most prominent increase was found at 50 mg CuNPs/L in 18-Elite line-1, 18-Elite line -3, and 18-Elite line-6 and 25 mg CuNPs/L in 18-Elite line-5. When treated with 50 mg CuNPs/L, the RL increased by 50, 153, and 33% in 18-Elite line-1, 18-Elite line-3, and 18-Elite line-6, respectively, and by 134 percent in 18-Elite line-5 when treated with 25 mg CuNPs/L. When compared to the control, the SL in 18-Elite line-1, 18-Elite line-3, and 18-Elite line-6 increased by 19, 20, and 37 percent, respectively, under 50 mg CuNPs/L, and by 54 percent in 18-Elite line-5 under 25 mg CuNPs/L. The FW also displayed the same results as for RL and SL, where a CuNPs concentration of 50 mg/L exhibited the most prominent effect on the FW of three wheat varieties. The maximum increase of 75, 95, and 35 in FW was observed for 18-Elite line-1, 18-Elite line -3, and 18-Elite line-6, respectively, treated with 50 mg CuNPs/L, while a maximum increase of 18% in FW was observed for 18-Elite line-5, treated with 25 mg CuNPs/L.

After 10 days of exposure, the CuNPs treatments modified the RL, SL, and FW of wheat. RL, SL, and FW were significantly increased, especially for varieties exposed to 50 mg CuNPs/L. These changes in the root and shoot system might be due to hormonal imbalances, like auxin and cytokinin imbalances [42]. Our findings are supported by the study [33], where CuNPs, at low concentration, increased the fresh weight of wheat. Moreover, we have observed that at 100 mg/L treatment of CuNPs, the seedling growth is significantly reduced compared to the treatment of 50 mg CuNPs/L, suggesting that a high dose of CuNPs can be lethal to plants. At high concentrations, CuNPs may release cupric ions that change the physiological processes of the plant. These modifications increase the capacity of particles that cross the cell membrane of a plant cell [3]. Previous studies also supported this fact that a higher concentration of CuNPs produces toxic effects, e.g., in chickpea and soybean, CuONPs increased root and shoot growth at 100 ppm, but beyond this concentration (200 ppm), the root and shoot growth was found to decrease [43]. Other studies also suggest the same result, e.g., CuNPs reduced the seedling length by 65 and 75% in *Phaseolus radiatus* and *Triticum aestivum*, respectively [44]. In a similar way, CuONPs decreased the RL in *Cucurbita pepo* when applied at a 1000 mg/L concentration [45].

### Effect of CuNPs on photosynthetic pigments of wheat seedling

**Fig 6:**
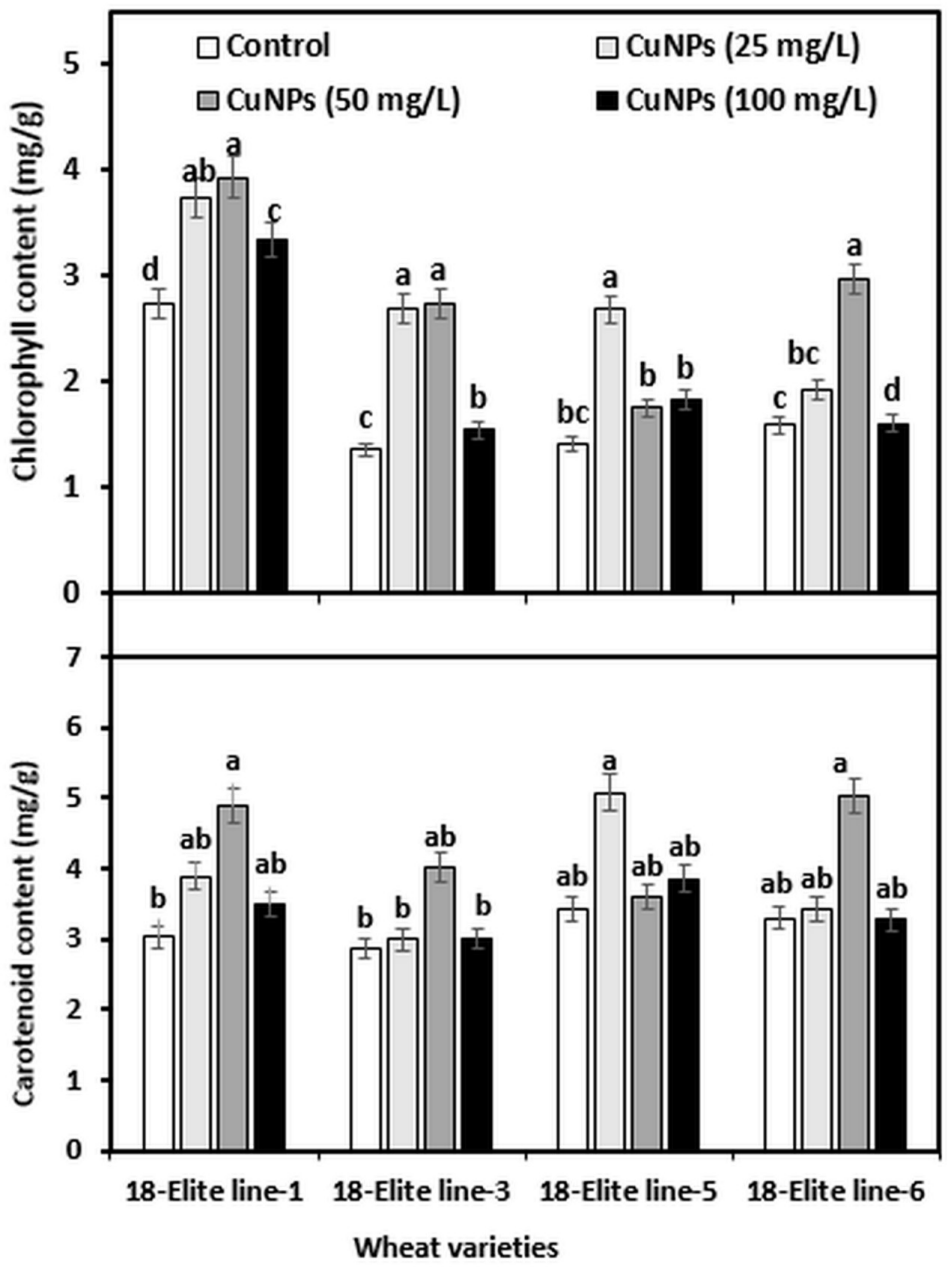
The total chlorophyll and carotenoid contents of 10 days wheat seedlings affected by different concentration of CuNPs. Different letters in the same sub-group of columns indicate significant difference at p < 0.05 level.

The effect of CuNPs on photosynthetic pigments of wheat varieties was measured in terms of total chlorophyll and carotenoid (Fig 6). Chlorophylls are located in the chloroplast where they play a crucial role in the photosynthesis system, which is highly related to plant productivity and biomass [46]. As a result, the total content of Chl and carotenoid in wheat varieties was measured in all treatments in the current study. The total chl content started increasing upon exposure to CuNPs and a higher level was found at 50 mg CuNPs/L in 18-Elite line-1, 18-Elite line -3, and 18-Elite line-6, while in 18-Elite line-5, 25 mg CuNPs/L evidenced stimulation of chl up to 48% as compared with no application of CuNPs (control). Carotenoid content in test plants also followed the same trend as total Chl. A maximum increase of 61, 40, and 42% in carotenoid content was found in 18-Elite line-1, 18-Elite line -3, and 18-Elite line-6, respectively, at 50 mg CuNPs/L. Some authors also noticed changes in the content of photosynthetic pigments as a result of nanoparticle application. For example, a concentration-dependent effect of CuNPs on chlorophyll and carotenoid pigments was studied by [47], where CuNPs increased the chlorophyll and carotenoid content. Similarly, *Vigna radiata* has shown increased chlorophyll and carotenoid content under the treatment of CuNPs [48]. We also observed a significant inhibitory effect at higher concentrations (100 mg CuNPs/L). This could be attributed directly to oxidative stress or the interaction of RuBP carboxylase, due to copper interaction with SH groups. Additionally, the Chl content decrease might also be due to the lowered shoot biomass upon contact with higher concentrations of CuONPs or to the membrane damage as a result of excess lipid peroxidation of chloroplast membranes under oxidative stress [49].

### Effect of CuNPs on phenolic and sugar content of wheat seedling

**Fig 7:**
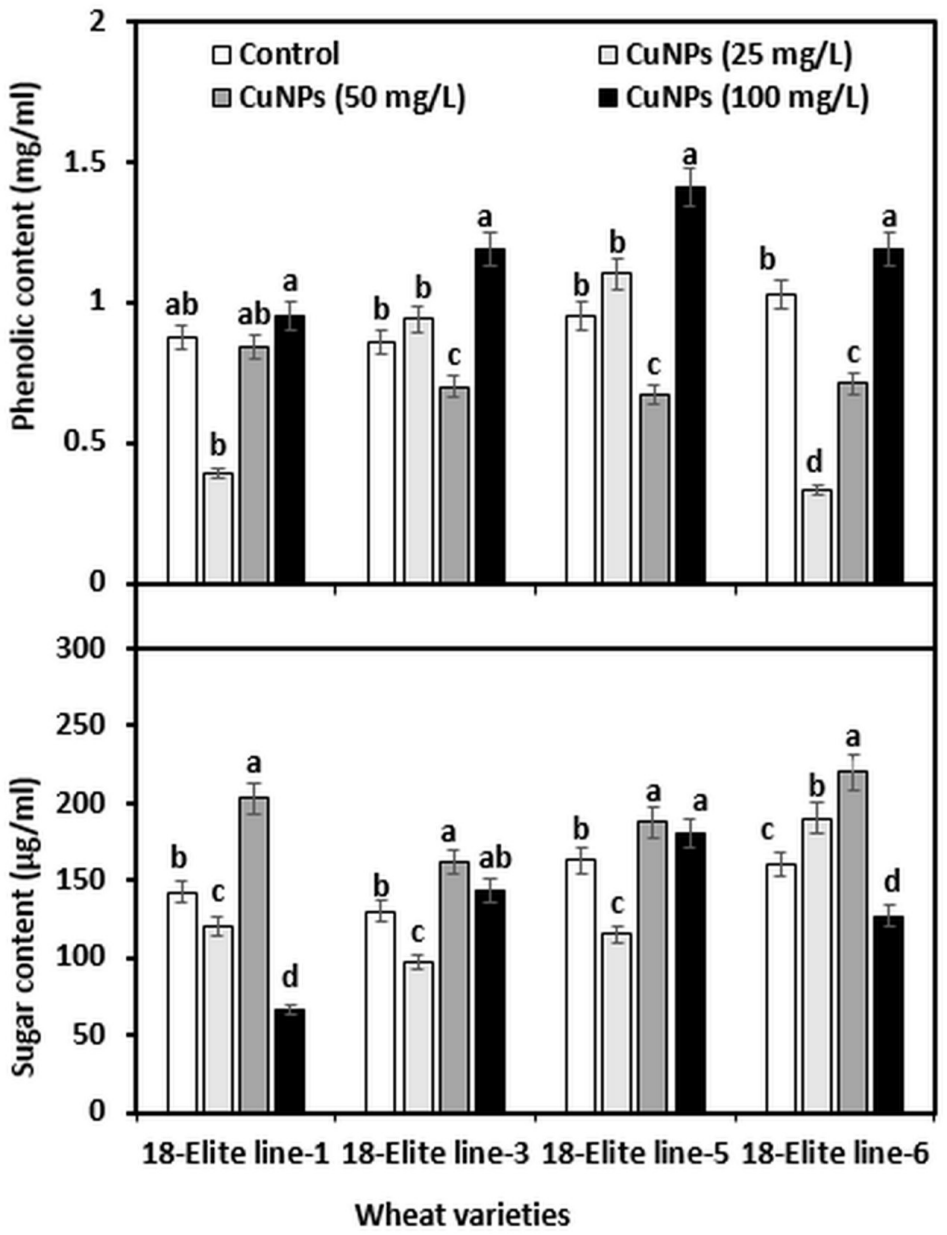
Total phenolic content and total soluble sugar content of 10 days wheat seedlings affected by different concentration of CuNPs. Different letters in the same sub-group of columns indicate significant difference at p < 0.05 level.

Treatment with CuNPs significantly influenced the content of phenol and soluble sugar in the seedlings of wheat (Fig 7). A maximum increase in total phenols was found at 100 mg CuNPs/L. It has been identified that plants induce the production of phenolic compounds in response to NPs [50]. In some other studies, an increase in phenolic content was also observed under a high concentration of nanoparticles. For example, fruits of *Jalapeno pepper* showed an increase in the total phenols under the application of CuNPs + Chitosan-PVA [51]. Similarly, the application of zinc nano fertilizer also increased the total phenols in pomegranate fruits [52]. This increase in phenolic content at higher concentrations may be related to the plant’s response to stress, as we observed 100 mg CuNPs/L caused toxic effects on wheat. Phenols are antioxidant compounds that trigger the synthesis of a series of secondary metabolites from the shikimic acid pathway or through phenylpropanoids under conditions of abiotic stress [53]. Therefore, the observed response may be related to ROS formation due to CuNPs. However, unlike the phenolic content, the soluble sugar content was found to be higher at 50 mg CuNPs/L treatment, as was the case with all other factors investigated. Overall, we found a positive effect of CuNPs on the growth and development of wheat. These results were supported by previous studies that showed Cu plays an important role in plant growth and development, and plant productivity [54].

## Conclusions

This study used an eco-friendly and appropriate method for the synthesis of CuNPs using *Mentha longifolia extract*. There were no chemical reagents or surfactant templates required, allowing green bioprocesses for a range of biomedical applications. During the application of CuNPs on wheat, we found in our study that with an increase in the concentration of CuNPs, the seed germination and growth of wheat plants were also increased. However, after a certain concentration (50 mg CuNPs/L), the seed germination and seedling growth were found to decrease. Overall results showed that application of CuNPs influences the seed germination and seedling growth of wheat at different concentrations. The higher germination and growth were found at 50 mg CuNPs/L treatment for 18-Elite line-1, 18-Elite line -3, and 18-Elite line-6 wheat varieties and at 25 mg CuNPs/L for 18-Elite line-5. Beyond this treatment, germination and growth were inhibited. Effective germination and growth at a certain optimum concentration and decreased germination and growth beyond this concentration may be attributed to the varieties of wheat and the uptake and accumulation of CuNPs by the roots, because the germination and growth were dependent on the exposure concentration of CuNPs. In particular, the contact of plants with NPs and the impact of such contact on plant growth could spur new research focus on nanobiotechnology. Further studies are needed to understand the mechanism of accumulation and uptake of CuNPs in plants and the way they act during seed germination and growth.

## Supporting information

S1 File (XLSX)

## References

[1] Fayaz AM, Balaji K, Girila M, Yadev R, Kalaichelvan PT, Venketesan R. Biogenic synthesis of silver nanoparticles and their synergistic effect with antibiotics: a study against gram-positive and gram-negative bacteria. Nanomed: Nanotechnol Biol Med. 2010;6(1):e103–e109. doi: 10.1016/j.nano.2009.04.006 19447203

[2] Gwinn MR, Vallyathan V. Nanoparticles: Health effects-pros and cons. Environ. Health Perspect. 2006;114(12):1818–1825. doi: 10.1289/ehp.8871 17185269 PMC1764161

[3] Singh A, Singh NB, Hussain I, Singh H, Singh SC. Plant nanoparticle interaction: an approach to improve agricultural practices and plant productivity. Int J Pharm Sci Invent. 2015; 4: 25–40.

[4] Buazar F. Impact of Biocompatible Nanosilica on Green Stabilization of Subgrade Soil. Sci Rep. 2019;9:15147. doi: 10.1038/s41598-019-51663-2 31641179 PMC6805850

[5] Moavi J, Buazar F, Sayahi MH. Algal magnetic nickel oxide nanocatalyst in accelerated synthesis of pyridopyrimidine derivatives. Sci Rep. 2021;11:6296. doi: 10.1038/s41598-021-85832-z 33739019 PMC7973480

[6] Sepahvand M, Buazar F, Sayahi MH. Novel marine-based gold nanocatalyst in solvent-free synthesis of polyhydroquinoline derivatives: Green and sustainable protocol. Applied Organomet Chem. 2020;34(12):e6000. doi: 10.1002/aoc.6000

[7] Hardani K, Buazar F, Ghanemi K, Kashisaz M, Baghlani-Nezhad MH, Khaledi-Naseb A, et al. Removal of toxic mercury (II) from water via Fe3O4/hydroxyapatite nanoadsorbent: an efficient, economic and rapid approach. AASCIT J Nanosci. 2015;1(1):11–18.

[8] Ghodake G, Seo YD, Lee DS. Hazardous phytotoxic nature of cobalt and zinc oxide nanoparticles assessed using *Allium cepa*. J Hazard Mater. 2011;186:952–955. doi: 10.1016/j.jhazmat.2010.11.018 21122986

[9] Ma Y, Kuang L, He X, Bai W, Ding Y, Zhand Z, et al. Effects of rare earth oxide nanoparticles on root elongation of plants. Chemosphere 2012;78:273–279. doi: 10.1016/j.chemosphere.2009.10.050 19897228

[10] Nekrasova GF, Ushakova OS, Ermakov AE, Uimin MA, Byzov IV. Effects of copper (II) ions and copper oxide nanoparticles on Elodea densa Planch. Russ J Ecol. 2011;42(6):458–463. doi: 10.1134/S1067413611060117

[11] Yruela I. Copper in plants. Braz J Plant Physiol. 2005;17(1):145–156. doi: 10.1590/S1677-04202005000100012

[12] Hong J, Rico C, Zhao L, Adeleye AS, Keller AA, Peralta-Videa JR, et al. Toxic effects of copper-based nanoparticles or compounds to lettuce (*Lactuca sativa*) and alfalfa (*Medicago sativa*). Environ Sci: Proc Imp. 2015;17:177–185. doi: 10.1039/C4EM00551A 25474419 PMC4326043

[13] White B, Yin M, Hall A, Le D, Stolbov S, Rahman T, et al. Complete CO oxidation over Cu_2_O nanoparticles supported on silica gel. Nano Lett. 2006;6(9):2095–2098. doi: 10.1021/nl061457v 16968032

[14] Buazar F, Cheshmehkani A, Kassaee MZ. Nanosteel synthesis via arc discharge: media and current effects. J Iran Chem Soc. 2012;9(2):151–156. doi: 10.1007/s13738-011-0038-3

[15] Kassaee MZ, Buazar F, Motamedi E. Effects of current on arc fabrication of Cu nanoparticles. J Nanomater. 2010;2010: 403197. doi: 10.1155/2010/403197

[16] Buazar F, Sweidi S, Badri M, Kroushawi F. Biofabrication of highly pure copper oxide nanoparticles using wheat seed extract and their catalytic activity: A mechanistic approach. Green Process Synth. 2019;8(1):691–702. doi: 10.1515/gps-2019-0040

[17] Keller AA, McFerran S, Lazareva A, Suh S. Global life cycle releases of engineered nanomaterials. J Nanopart Res. 2013;15:1692. doi: 10.1007/s11051-013-1692-4

[18] Murugan K, Senthilkumar B, Senbagam D, Al-Sohaibani S. Biosynthesis of silver nanoparticles using *Acacia leucophloea* extract and their antibacterial activity. Int J Nanomed. 2014;9:2431–2438. doi: 10.2147/IJN.S61779 24876776 PMC4035312

[19] Mohammed SA, Khashan KS, Jabir MS, Abdulameer FA, Sulaiman GM, Al-Omar MS, et al. Copper Oxide Nanoparticle-Decorated Carbon Nanoparticle Composite Colloidal Preparation through Laser Ablation for Antimicrobial and Antiproliferative Actions against Breast Cancer Cell Line, MCF-7. BioMed Res Int. 2022;2022:9863616. doi: 10.1155/2022/9863616 35299896 PMC8923787

[20] Koopi H, Buazar F. A novel one-pot biosynthesis of pure alpha aluminum oxide nanoparticles using the macroalgae *Sargassum ilicifolium*: a green marine approach. Ceramics Int. 2018;44(8):8940–8945. doi: 10.1016/j.ceramint.2018.02.091

[21] Safat S, Buazar F, Albukhaty S, Matroodi S. Enhanced sunlight photocatalytic activity and biosafety of marine-driven synthesized cerium oxide nanoparticles. Sci Rep. 2021;11(1):1–11. 34282244 10.1038/s41598-021-94327-w PMC8289931

[22] Buazar F, Baghlani-Nejazd MH, Badri M, Kashisaz M, Khaledi-Nasab A, Kroushawi F. Facile one-pot phytosynthesis of magnetic nanoparticles using potato extract and their catalytic activity. Starch-Stärke. 2016;68(7–8):796–804. doi: 10.1002/star.201500347

[23] Buazar F, Bavi M, Kroushawi F, Halvani M, Khaledi-Nasab A, Hossieni SA. Potato extract as reducing agent and stabiliser in a facile green one-step synthesis of ZnO nanoparticles. J Exper Nanosci. 2016;11(3):175–184. doi: 10.1080/17458080.2015.1039610

[24] Gholizadeh BS, Buazar F, Hosseini SM, Mousavi SM. Enhanced antibacterial activity, mechanical and physical properties of alginate/hydroxyapatite bionanocomposite film. Int J Biolog Macromol. 2018;116:786–792. doi: 10.1016/j.ijbiomac.2018.05.104 29777815

[25] Rezazadeh NH, Buazar F, Matroodi S. Synergistic effects of combinatorial chitosan and polyphenol biomolecules on enhanced antibacterial activity of biofunctionalized silver nanoparticles. Sci Rep. 2020;10(1):1–13. 33184403 10.1038/s41598-020-76726-7 PMC7665213

[26] Kim BS, Song JY. Biological synthesis of metal nanoparticles. In: Hou CT, Shaw JF, editors. Biocatalysis and Agricultural Biotechnology. CRC Press, Boca Raton; 2009. pp. 399–407.

[27] Shankar SS, Rai A, Ahmad A, Sastry M. Rapid synthesis of Au, Ag, and bimetallic Au core–Ag shell nanoparticles using Neem (*Azadirachta indica*) leaf broth. J Coll Interf Sci. 2004;275:496–502. doi: 10.1016/j.jcis.2004.03.003 15178278

[28] Faruqee R, Coleman JR, Scott T. Managing price risk in the Pakistan wheat market. World Bank Econ Rev. 1997;11(2):263–269. doi: 10.1093/wber/11.2.263

[29] Hafeez A, Razzaq A, Mahmood T, Jhanzab HM. Potential of copper nanoparticles to increase growth and yield of wheat. J Nanosci Adv Technol. 2015;1:6e11. doi: 10.24218/jnat.2015.02

[30] Zhang Z, Ke M, Qu Q, Peijnenburg WJGM, Lu T, Zhang Q, et al. Impact of copper nanoparticles and ionic copper exposure on wheat (*Triticum aestivum* L.) root morphology and antioxidant response. Environ Pollut. 2018;239:689e697. doi: 10.1016/j.envpol.2018.04.066 29715688

[31] Lichtenthaler HK. Chlorophylls and carotenoids: Pigments of photosynthetic biomembranes. Methods Enzymol. 1987;148:350–382. doi: 10.1016/0076-6879(87)48036-1

[32] Pourmorad F, Hosseinimehr SJ, Shahabimajd N. Antioxidant activity, phenol and flavonoid contents of some selected Iranian medicinal plants. Afr J Biotechnol. 2006;5(11):1142–1145.

[33] Bhanushali MP, Jaybhaye SV, Gutte AV. Copper nanoparticles using onion (*Allium cepa*) extract and their application in plant growth. Int J Life Sci. 2017;5(4):661–666.

[34] Valli G, Suganya M. Green synthesis of copper nanoparticles using *Cassia fistula* flower extract. J Bio Innov. 2015;4(5):162–170.

[35] Prema P. Chemical mediated synthesis of silver nanoparticles and its potential antibacterial application. Anal Mod Technol App. 2010;2010:151–166.

[36] Jana S, Pal T. Synthesis, characterization and catalytic application of silver nanoshell coated functionalized polystyrene beads. J Nanosci Nanotechnol. 2007;7:2151–2156. doi: 10.1166/jnn.2007.785 17655008

[37] Zhou D, Abdel-Fattah AI, Keller AA. Clay particles destabilize engineered nanoparticles in aqueous environments. Environ Sci Technol. 2012;46(14):7520–7526. doi: 10.1021/es3004427 22721423

[38] Sulaiman GM, Tawfeeq AT, Jaaffer MD. Biogenic synthesis of copper oxide nanoparticles using olea europaea leaf extract and evaluation of their toxicity activities: An in vivo and in vitro study. Biotechnol Prog. 2018;34(1):218–230. doi: 10.1002/btpr.2568 28960911

[39] Shah V, Belozerova L. Influence of metal nanoparticles on the soil microbial community and germination of lettuce seeds. Water Air Soil Poll. 2009;197(1–4):143–148. doi: 10.1007/s11270-008-9797-6

[40] Khodakovskaya MV, Silva KD, Biris AS, Dervishi E, Villagarcia H. Carbon nanotubes induce growth enhancement of tobacco cells. ACS Nano 2012;6(3):2128–2135. doi: 10.1021/nn204643g 22360840

[41] Zheng L, Hong F, Lu S, Liu C, Effect of nano-TiO_2_ on strength of naturally aged seeds and growth of spinach. Biol Trac Elem Res. 2005;104(1):83–91. doi: 10.1385/BTER:104:1:083 15851835

[42] Yuan HM, Liu WC, Jin Y, Lu YT. Role of ROS and auxin in plant response to metal mediated stress. Plant Sig Behav. 2013;8:e24671. doi: 10.4161/psb.24671 23603941 PMC3906317

[43] Adhikari T, Kundu S, Biswas AK, Tarafdar JC, Rao AS. Effect of copper oxide nano particle on seed germination of selected crops. J Agri Sci Technol. 2012;2:815–823.

[44] Lee W, An Y, Yoon H, Kweon H. Toxicity and bioavailability of copper nanoparticles to the terrestrial plants mung bean (*Phaseolus radiatus*) and wheat (*Triticum aestivum*): Plant agar test for water-insoluble nanoparticles. Environ Toxicol Chem. 2008;27(9):1915–1921. doi: 10.1897/07-481.1 19086317

[45] Stampoulis B, Sinha SK, White JC. Assay-dependent phytotoxicity of nanoparticles to plants. Environ Sci Technol. 2009;43:9473–9479. doi: 10.1021/es901695c 19924897

[46] Tamez C, Morelius EW, Hernandez-Viezcas JA, Peralta-Videa JR, Gardea-Torresdey J. Biochemical and physiological effects of copper compounds/nanoparticles on sugarcane (*Saccharum officinarum*). Sci Total Environ. 2019;649:554–562. doi: 10.1016/j.scitotenv.2018.08.337 30176466

[47] Pradhan S, Patra P, Mitra S, Dey KK, Basu S, Chandra S, et al. Copper nanoparticle (CuNP) nanochain arrays with a reduced toxicity response: a biophysical and biochemical outlook on *Vigna radiata*. J Agri Food Chem. 2015;63:2606–2617. doi: 10.1021/jf504614w 25686266

[48] Yamasaki H, Pilon M, Shikanai T. How do plants respond to copper deficiency? Plant Sig Behav. 2008;3(4):231–2. doi: 10.4161/psb.3.4.5094 19704637 PMC2634185

[49] Halliwell B, Gutteridge JMC. Free radicals in biology and medicine. Clarendon Press, Oxford; 1989.

[50] Zafar H, Ali A, Ali JS, Haq IU, Zia M. Effect of ZnO nanoparticles on *Brassica nigra* seedlings and stem explants: growth dynamics and antioxidative response. Front Plant Sci. 2016;7:535. doi: 10.3389/fpls.2016.00535 27148347 PMC4837972

[51] Pinedo-Guerrero ZH, Hernández-Fuentes A, Ortega-Ortiz H, Benavides-Mendoza A, Cadenas-Pliego G, Juárez-Maldonado A. Cu nanoparticles in hydrogels of chitosan-PVA affects the characteristics of post-harvest and bioactive compounds of Jalapeno pepper. Molecules 2017;l 22:926. doi: 10.3390/molecules22060926 28574445 PMC6152709

[52] Davarpanah S, Tehranifar A, Davarynejad G, Abadía J, Khorasani R. Effects of foliar applications of zinc and boron nano-fertilizers on pomegranate (*Punica granatum* cv. Ardestani) fruit yield and quality. Sci Hortic. 2016;210:57–64. doi: 10.1016/j.scienta.2016.07.003

[53] Swieca M. Hydrogen peroxide treatment and the phenylpropanoid pathway precursors feeding improve phenolics and antioxidant capacity of *Quinoa Sprouts* via an induction of L-tyrosine and L-phenylalanine ammonia-lyases activities. J Chem. 2016;2016:1–7. doi: 10.1155/2016/1936516

[54] Xie X, He Z, Chen N, Tang Z, Wang Q, Cai Y. The roles of environmental factors in regulation of oxidative stress in plant. Biomed Res Int. 2019;2019:9732325. doi: 10.1155/2019/9732325 31205950 PMC6530150

